# Bypassing primary healthcare facilities for maternal healthcare in North West Ghana: socio-economic correlates and financial implications

**DOI:** 10.1186/s12913-021-06573-3

**Published:** 2021-06-02

**Authors:** Valeria Avoayea Amoro, Gilbert Abotisem Abiiro, Kennedy A. Alatinga

**Affiliations:** 1 Upper West Regional Coordinating Council, Local Government Service, Wa, Ghana; 2 Department of Health Services, Policy, Planning, Management and Economics, School of Public Health, University for Development Studies, Tamale, Ghana; 3 Department of Planning, Faculty of Planning and Land Management, Simon Diedong Dombo University of Business and Integrated Development Studies, Wa, Ghana; 4 Department of Community Development, Faculty of Planning and Land Management, Simon Diedong Dombo University of Business and Integrated Development Studies, Wa, Ghana

**Keywords:** Primary Health Care, Maternal healthcare, bypassing, socio-economic correlates, financial cost, Ghana

## Abstract

**Background:**

Bypassing primary health care (PHC) facilities for maternal health care is an increasing phenomenon. In Ghana, however, there is a dearth of systematic evidence on bypassing PHC facilities for maternal healthcare. This study investigated the prevalence of bypassing PHC facilities for maternal healthcare, and the socio-economic factors and financial costs associated with bypassing PHC facilities within two municipalities in Northwestern Ghana.

**Methods:**

A quantitative cross-sectional design was implemented between December 2019 and March 2020. Multistage stratified sampling was used to select 385 mothers receiving postnatal care in health facilities for a survey. Using STATA 12 software, bivariate analysis with chi-square test and binary logistic regression models were run to determine the socio-economic and demographic factors associated with bypassing PHC facilities. The two-sample independent group t-test was used to estimate the mean differences in healthcare costs of those who bypassed their PHC facilities and those who did not.

**Results:**

The results revealed the prevalence of bypassing PHC facilities as 19.35 % for antenatal care, 33.33 % for delivery, and 38.44 % for postnatal care. The municipality of residence, ethnicity, tertiary education, pregnancy complications, means of transport, nature of the residential location, days after childbirth, age, and income were statistically significantly (*p* < 0.05) associated with bypassing PHC facilities for various maternal care services. Compared to the non-bypassers, the bypassers incurred a statistically significantly (*P* < 0.001) higher mean extra financial cost of GH₵112.09 (US$19.73) for delivery, GH₵44.61 (US$7.85) for postnatal care and ₵43.34 (US$7.65) for antenatal care. This average extra expenditure was incurred on transportation, feeding, accommodation, medicine, and other non-receipted expenses.

**Conclusions:**

The study found evidence of bypassing PHC facilities for maternal healthcare. Addressing this phenomenon of bypassing and its associated cost, will require effective policy reforms aimed at strengthening the service delivery capacities of PHC facilities. We recommend that the Ministry of Health and Ghana Health Service should embark on stakeholder engagement and sensitization campaigns on the financial consequences of bypassing PHC facilities for maternal health care. Future research, outside healthcare facility settings, is also required to understand the specific supply-side factors influencing bypassing of PHC facilities for maternal healthcare within the study area.

## Background

Bypassing primary health care (PHC) facilities for maternal health care is an increasing phenomenon within low- and middle-income countries (LMICs) [1–4]. Bypassing for maternal health care is the act of mothers and prospective mothers choosing to seek care from a facility that is farther from their homes of residence whiles ignoring their immediate healthcare facilities that have been officially permitted to provide such services [3, 5, 6]. This practice has implications for the effective and equitable delivery of various maternal health care services such as antenatal care (ANC), delivery and postnatal care (PNC) [6, 7]. Bypassing can lead to overcrowding at the facility where care is sought while the bypassed facility is being underutilized [6, 8–10].

In line with the global priority on maternal health care, the Government of Ghana has since 2008, implemented a free maternal health care policy within its National Health Insurance Scheme (NHIS) [11]. The NHIS policy exempts pregnant women from paying registration fees and health insurance premiums before being registered with the scheme. Pregnant women registered with the NHIS, are entitled to free services including ANC, delivery, PNC and childcare services within the period of pregnancy and three months after child birth at any NHIS accredited facility [12]. However, some empirical evidence reveals the persistence of out-of-pocket payments for maternal care in Ghana [13].

In Ghana, healthcare seekers, including that of maternal healthcare seekers, are required to first visit their PHC facilities, which are the basic health care facilities located close to them, to seek care and depending on the severity of the condition, referrals are made as and when needed for the onward continuation of care at higher level health care facilities [14, 15]. The Community-based Health Planning and Services (CHPS) zones at the community level and health centers at the sub-district level form the basic levels of PHC [16]. CHPS is supposed to be equipped to provide ANC and PNC services but only where a midwife is present that spontaneous vaginal delivery services can also be provided [16]. In the absence of a midwife, the PHC facilities can only carry out emergency delivery but caesarean sections are only provided at the higher-level facilities where a medical doctor is available. These PHC facilities serve as gatekeepers within the health system [14]. However, enforcing proper gatekeeper and referral systems in Ghana and within other African contexts is practically challenging [17]. Roder-Dewan et al. [18] even seem to question the rationale for a gatekeeper system for maternal healthcare in their recent call for redesigning healthcare models in LMICs to shift all child births to higher level facilities such as hospitals. The call attributed global inequities in maternal health outcomes to current healthcare models requiring that substantial deliveries be made at PHC facilities and argues that emerging evidence demonstrates that such PHC facilities lack the capacity to provide quality basic emergency obstetric and newborn care[18–21]. However, Hanson et al. [22] argued that local needs, contextual peculiarities, human resource availability and the need for a strengthened local health system should determine the fitness of recommended healthcare models. It is therefore not clear whether bypassing, especially as pertains to deliveries, is indeed a problem or a necessity. What is clear is that in LMICs, it is very common for healthcare seekers to move straight to the district, regional and teaching hospitals to seek care without any point of contact with their PHC facilities [14].

In Asia, Nepal records an average bypassing rate of 50 % [4, 23] and that of India was reported as 37.7 % [6] or 38.9 % [10] for various maternal care services. In sub-Saharan Africa (SSA), the prevalence of bypassing health facilities for maternal care varies from one setting to another. It ranges between 76 and 40 % in Tanzania [3, 24, 25]; 60 and 46 % in Kenya [5] to 30.8 % in Mozambique [26]. In line with the Andersen behavioural model, a number of socio-economic factors [27, 28] predispose women to the phenomenon of bypassing [29]. These include maternal age, educational status, income, experience of complications during pregnancy, residential location, means of transport and distance to health facilities among others [2, 4, 5, 10, 10, 23–26, 30–32]. Bypassing is also reported to be associated with an extra cost in terms of transportation, feeding, accommodation, consultation, medication and opportunity cost [2, 3, 23–25, 31]. Living expenses of both the maternal care seeker and the accompanying family member(s) to the preferred facility can be very high thus making the households of those who bypassed their PHC facilities to spend more resources on health care compared to their counterparts who did not bypass [23–25].

However, this existing evidence on bypassing healthcare facilities for maternal healthcare is only reported in a few countries such as Tanzania, Kenya and Mozambique in SSA and Nepal and India in Asia. To the best of our knowledge, the only study that explicitly examined the phenomenon of bypassing in Ghana is the one by Yaffee et al. [32]. Yaffee et al.[32] found that 33.9 % of the people seeking care at the Accident and Emergency Care Unit of the Komfo Anokye Teaching Hospital in Ghana had bypassed their nearest PHC facilities. This study did not focus on maternal healthcare. To provide evidence to enhance our understanding of existing inequities in the use of healthcare facilities for maternal health care in Ghana, our study assessed the prevalence of bypassing PHC facilities for maternal care, and the socio-economic factors and financial costs associated with the phenomenon in the Wa and Jirapa municipalities of the Upper West Region of Ghana.

## Methods

### Study setting

The Upper West Region is in the northwestern part of Ghana. The region has a total population of 702,110 of which 360,928 (51.4 %) are females and 46.1 % of the female population is within the reproductive age range of 15–49 [33]. The total fertility rate of the region is 3.5 [34]. According to the Upper West Regional Health Directorate, Ghana Health Service (UWRHD-GHS) [35] as at the end of 2018, the region had 375 health facilities. In 2018, the region recorded 27,032 ANC and 23,458 PNC attendance, 69 % skilled deliveries and an institutional maternal mortality of 22 deaths (96.4 per 100,000 live births) [35]. Wa Municipality is the oldest municipality in the region and doubles as the regional capital. The total population of the Municipality is 107,214, representing 15.3 % of the regional population. Females constitute 50.6 % of which 29, 396 are within the child bearing ages (15–49 years) with a total fertility rate of 3.3 [34]. Apart from the regional hospital, Wa Municipality is host to a total of 50 other health facilities, comprising seven health centers, 26 CHPS compounds, eight clinics, three hospitals, one maternity home and five private health facilities [35]. The total registrants for ANC in 2018 in the Wa Municipality was 6,436 and that of PNC was 6, 382 [35].

According to the Ghana Statistical Service[33], the Jirapa Municipality has a total population of 88,402 which represents 12.6 % of the regional population. The females represent 53 % of the population and women within the childbearing ages of 15–49 are 20,604 with a total fertility rate of 3.5 %. The St Joseph hospital, a Christian Health Association of Ghana facility, is the only hospital in the Municipality and serves as the municipal hospital. The other health facilities in the Municipality include: one polyclinic, seven health centres, and 22 CHPS compounds [35]. In 2018, the Municipality recorded ANC registration of 2,398 and 2,406 PNC registrations with skilled delivery rate of 57 % [35]. The two municipalities were therefore purposively selected to reflect the heterogeneity in distribution of health facilities and respondents’ characteristics.

### Study design and sampling

A cross-sectional design was employed to gather quantitative data from newly delivered mothers who were still within their postpartum period (not more than six weeks after delivery) and were visiting health facilities in the Wa and Jirapa Municipalities for PNC. Those mothers who delivered within the past six weeks were specifically targeted to represent maternal health care seekers because since they were in their last stage of the maternal health care seeking (postnatal) process, they would have already visited healthcare facilities for ANC and delivery and could therefore provide information on all the three forms of maternal healthcare services. The study used Creswell’s [36] formula to compute the sample size as follows;

$$n={t}^{2 }*p (1-p)/{m}^{2}$$.

Where; *n* = the required sample size; t = confidence level at 95 % with standard deviation of 1.96; *p* = the estimated prevalence of the variable of interest (we assumed 55 % prevalence of bypassing PHC facilities for maternal care based on an average of the range of 40 and 70 % prevalence values reported in SSA); m = margin of error at 5 % with standard value of 0.05.

Substituting into the formula;

$$n={1.96}^{2 }*0.55 (1-0.55)/{0.05}^{2}$$

Therefore, *n* = 380.

In a bid to cater for non-response, the sample size was increased by about 2.5 % to 390. This sample size was proportionally allocated between the two municipalities based on their respective number of PNC registrations; 72.6 % for Wa and 27.4 % for Jirapa.

A multistage stratified sampling technique was used to select the respondents. Firstly, the study area was stratified into urban (Wa Municipality) and rural (Jiripa Municipality) strata. Within each of the municipalities, the existing health care facilities were further stratified into three, based on the type/level of the facility. The three facility-based strata comprised: The municipal hospitals, sub-municipal health centers and CHPS compounds. Within each municipality, the municipal hospital was purposively selected. In addition, two sub-municipal health centers and two CHPS compounds from each municipality were selected through simple random sampling. In all, two municipal hospitals, four sub-district health centers and four CHPS compounds were selected for the study. Within each of the selected facilities, PNC service seekers were systematically sampled as they arrived and registered for PNC service at each facility to constitute the final study sample. The daily attendance registers for PNC services in each facility constituted the sampling frame.

### Data collection and analysis

An institutional-based survey was used for the data collection. A structured questionnaire was administered to PNC seekers in the form of exit interviews. To enhance the validity and reliability of the questionnaire, it was designed with reference to already tested and validated questionnaires used to collect data within the Ghanaian context. Appropriate questions were adopted from the Ghana Demographic and Health Survey and the Ghana Living Standard Survey questionnaires. The questionnaire elicited responses on the socio-economic, demographic, and health-related characteristics of the respondents, PHC facilities, use of maternal health care services, choice of health facilities for maternal care and the reasons behind the facility choices etc. The costs associated with the use of each facility for each of the maternal health services over the period of pregnancy, childbirth and postnatal were also elicited. The questionnaires were administered by four trained research assistants under the supervision of the first author using mobile phones between December 2019 and March 2020. The questionnaire was administered in both English (to literate mothers who preferred the English language) and the two dominant local languages (Wale and Dagaare) in the study area. The research assistants were fluent in all three languages. Each questionnaire was administered within 40 min. All methods were carried out in accordance with the principles of the Declaration of Helsinki.

The data was analyzed using STATA 12 software. Descriptive statistics (percentages, means/medians, standard deviations/ranges) for the respondent’s socio-economic and demographic characteristics and the prevalence of bypassing PHC facilities were generated. The prevalence of bypassing was calculated as the proportion of women who received each of the maternal care services from distant health facilities meanwhile there were closest health facilities that were officially permitted by GHS to provide that type of maternal care service and yet the women were not referred by these closest PHC facilities to the distant health facilities. Bivariate analysis (cross-tabulations and chi-square test of significance) and binary logistic regression modelling were also done to identify the socio-economic factors that were associated with bypassing of PHC facilities for the various maternal healthcare services. Bypassing was coded as a dichotomous variable and constituted the dependent variable for the regression analysis while all the socio-economic variables that showed significant relationships (age, income, educational attainment, complications during pregnancy, municipality of residence, ownership of means of transport, among others) with bypassing in the bivariate analysis were used as the independent variables. Statistical significance for all the analyses were determined at the 95 % confidence interval. Robust standard errors were generated in the model estimation. The average cost of bypassing PHC facilities was determined using the two-sample independent group t-test of differences in means between those who bypassed their PHC facilities and those who did not.

## Results

### Socio-economic and demographic characteristics of the respondents

Of the 390 sampled mothers, 385 responded to the study, representing a response rate of 98.7 %. All the 385 respondents received PNC, 372 (96.62 %) received ANC for their current pregnancy and 381 (98.96 %) delivered at a health facility. As shown in Table 1, majority (72.21 %) of the respondents were from the Wa Municipality, 56.10 % lived in rural areas and 94.03 % were married. Dagaaba constituted the largest ethnic group (48.83 %) of the respondents. The proportion of Christians (48.05 %) is slightly higher than that of the Muslims (45.71 %) while the rest (6.23 %) practiced Traditional African Religion. 25.97 % of the mothers never had any formal education with the highest level of educational attainment (21.56 %) being Junior High School (JHS) while 18.96 % obtained tertiary level education. The average age (both mean and median) was 28 years. Majority (49.09 %) of the mothers were within the childbearing age category of 26–35 years. The highest proportion (26.49 %) of the mothers were unemployed. For those employed, the informal sector was the main source of employment as many of them were employed in vocational activities (23.12 %), farming (20.00 %) and trading (15.06 %). Only 15.32 % of the mothers were employed in the formal sector as teachers. 23.38 % of the mothers reported not earning any income monthly. The mean monthly income for the mothers was GH₵347.51(US$61.18 [1US$= GH₵5.68]) and the median was GH₵100(US$17.60). Following the minimum national monthly wage rate of about GH₵354 (US$62.32) or 11.82 (US$2.08) per day [37], about 70 % of the mothers’ income fell below the national minimum wage rate. 47 % of the respondents owned personal motorbikes or cars. Majority of the respondents (68.57 %) had a previous history of pregnancy before their most recent birth, with 13.25 % of them reporting a pregnancy-related complication with this previous pregnancy. The average number of livebirths per woman was about two. Generally, majority of the women rated their health status during the pregnancy as good with very few reporting poor health. The study reports very high rates of health insurance coverage among the mothers before (92.99 %) and during (97.14 %) their recent pregnancies.

**Table 1:** Socio-Economic and Demographic Characteristics of the Mothers.

Characteristic	Categories	Number of respondents	Percentage
Municipality of residence	Jirapa	107	27.79
	Wa	278	72.21
Nature of residential location	Urban	107	27.79
	Peri-urban	62	16.10
	Rural	216	56.10
Age	16–25	141	36.62
	26–35	189	49.09
	> 35	55	14.29
Religion	Christian	185	48.05
	Islam	176	45.71
	Traditional/others	24	6.23
Ethnicity	Waala	114	29.61
	Dagaaba	188	48.83
	Others	83	21.56
Current marital status	Not Married	23	5.97
	Married	362	94.03
Education	No formal education	100	25.97
	Primary	61	15.84
	JHS	83	21.56
	SHS	68	17.66
	Tertiary	73	18.96
Occupation	Formal sector employee	59	15.32
	Vocation	89	23.12
	Trade	58	15.06
	Farming	77	20.00
	Unemployed	102	26.49
Monthly Mother’s income (GH₵)	Zero	90	23.38
	10–100	107	27.79
	120–350	73	18.96
	370–900	59	15.32
	1000–3000	56	14.55
Ownership of Car/Motorbike	Yes	181	47.01
Previous history of pregnancy	Yes	264	68.57
Complications during previous pregnancy(cies)	Yes	51	13.25
Days after current birth	0-5days	70	18.18
	6-10days	189	49.09
	11–39 days	63	16.36
	40 and more days	63	16.36
Complications with recent pregnancy	Yes	46	11.95
Self-rated health status during recent pregnancy	Excellent	30	7.79
	Very good	142	36.88
	Good	173	44.94
	Poor	40	10.39
Parity	One Child	146	37.92
	Two children	94	24.42
	Three Children	76	19.74
	Four-Eight	69	17.92
Total		385	100

### Prevalence of bypassing PHC facilities for maternal health care

As shown in Table 2, the prevalence of bypassing PHC facilities for ANC at least once is 51.08 % but 19.35 % for all ANC visits throughout the course of the pregnancy. This implies that 19.35 % of the mothers who received ANC never visited their PHC facilities for the service. This number does not include the 7 (1.88 %) mothers who reported that they were referred to their non-PHC facilities for ANC. Although 56.43 % of the mothers did not deliver at their PHC facilities, 23.10 % out of this figure were referred by health care professionals for delivery at their non-PHC facilities. In effect, 33.33 % of those who delivered at health care facilities bypassed their PHC facilities. PNC recorded the highest prevalence of PHC facility bypassing with 38.44 % of the mothers receiving PNC outside their PHC facilities without referrals from healthcare providers. Only one mother reported being referred to a non-PHC facility for PNC.

**Table 2:** Prevalence of Bypassing PHC Facilities for Maternal Care.

Variable	ANC		Delivery		PNC	
	Number	Percent	Number	Percent	Number	percent
Facility-based care	372	96.62	381	98.96	385	100
Non-use of PHC facility	197	52.96	215	56.43	149	38.7
Referred to non-PHC facility	7	1.88	88	23.10	1	0.26
Bypassed PHC in at least one visit	190	51.08				
Bypassed PHC in all the visits	72	19.35	127	33.33	148	38.44

### Association between socio-economic and demographic characteristics of respondents and bypassing of PHC facilities for maternal health care

 Table 3 presents the exploratory bivariate analysis of the associations between socio-economic and demographic characteristics and bypassing of PHC facilities for maternal health care. As shown in Table 3, the bivariate associations between PHC bypassing and some socio-economic variables; including religion, marital status, previous history of pregnancy and its complications, parity, and self-rated health status; were not statistically significant (*p* > 0.05) for ANC, delivery, and PNC. Those variables were therefore excluded from the multivariate regression modelling.

**Table 3:** Bivariate Analysis of the Association Between Mothers’ Characteristics and Bypassing of PHC Facilities for ANC.

Characteristic	Categories	ANC: Number (%)				Delivery: Number (%)				PNC: Number (%)			
		Bypassed	Not bypassed	X^2^	Pr	Bypassed	Not bypassed	X^2^	Pr	Bypassed	Not bypassed	X^2^	Pr
Municipality of residence	Jirapa	3(3)	97 (97)	23.44	0.000	7(6.54)	100(93.46)	48.057	0.000	5(4.67)	102(95.33)	71.41	0.000
	Wa	69(25.37)	203(74.63)			120(43.80)	154(56.20)			143(51.44)	135(48.56)		
Nature of residential location	Urban	11(10.28)	96(89.72)	14.69	0.001	27(25.96)	77(74.04)	19.54	0.000	34(31.78)	73(68.22)	9.01	0.011
	Peri-urban	7(11.48)	54 (88.52)			9(14.75)	52(85.25)			17(27.42)	45(72.58)		
	Rural	54(26.47)	150(73.53)			91(42.13)	125(57.87)			97(44.91)	119(55.09)		
Age	16–25	24(17.39)	114(82.61)	6.55	0.038	35(25.36)	103(74.64)	8.41	0.015	38(26.95)	103(73.05)	14.17	0.001
	26–35	43(24.02)	136(75.98)			67(35.45)	122(64.55)			81(42.86)	108(57.14)		
	> 35	5 (9.09)	50(90.91)			25(46.30)	29(53.70)			29(52.73)	26(47.27)		
Religion	Christian	39(21.91)	139(78.09)	4.32	0.115	63(34.24)	121(65.76)	0.1402	0.932	80(43.24)	105(56.76)	4.1472	0.126
	Islam	32(18.82)	138(81.18)			56(32.37)	117(67.63)			58(32.95)	118(67.05)		
	Traditional/others	1(4.17)	23(95.83)			8(33.33)	16(66.67)			10(41.67)	14(58.33)		
Ethnicity	Waala	23(20.91)	87(79.09)	2.31	0.314	27(24.11)	85(75.89)	25.08	0.000	30(26.32)	84(73.68)	39.01	0.000
	Dagaaba	30(16.39)	153(83.61)			54(28.88)	133(71.12)			62(32.98)	126(67.02)		
	Others	19(24.05)	60(75.95)			46(56.10)	36(43.90)			56(67.47)	27(32.53)		
Current marital status	Not Married	1(4.76)	20(95.24)	3.036	0.081	5(21.74)	18(78.26)	1.4807	0.224	7(30.43)	16(69.57)	0.66	0.416
	Married	71(20.23)	280(79.77)			122(34.08)	236(65.92)			141(38.95)	221(61.05)		
Education	No formal	9(9.09)	90(90.91)	27.94	0.000	26(26.53)	72(73.47)	21.36	0.000	33(33.00)	67(67.00)	31.42	0.000
	Primary	4(6.90)	54(93.10)			10(16.67)	50(83.33)			11(18.03)	50(81.97)		
	JHS	16(19.75)	65(80.25)			25(30.49)	57(69.51)			26(31.33)	57(68.67)		
	SHS	18(27.69)	47(72.31)			32(47.06)	36(52.94)			40(58.82)	28(41.18)		
	Tertiary	25(36.23)	44(63.77)			34(46.58)	39(53.42)			38(52.05)	35(47.95)		
Occupation	Formal worker	17(29.82)	40(70.18)	17.13	0.002	29(49.15)	30(50.85)	19.30	0.001	32(54.24)	27(45.76)	21.20	0.000
	Vocation	21(24.14)	66(75.86)			36(40.45)	53(59.55			43(48.31)	46(51.69)		
	Trade	10(18.52)	44(81.48)			22(37.93)	36(62.07)			23(39.66)	35(60.34)		
	Farming	3(3.95)	73(96.05)			13(17.81)	60(82.19			16(20.78)	61(79.22)		
	Unemployed	21(21.43)	77(78.57)			27(26.47)	75(73.53)			34(33.33)	68(66.67)		
Monthly Mother’s income (GH₵)	Zero	18(20.93)	68(79.07)	9.44	0.051	23(25.84)	66(74.16)	19.58	0.001	33(36.67)	57(63.33)	18.29	0.001
	10–100	13(12.38)	92(87.62)			24(22.86)	81(77.14)			25(23.36)	82(76.64)		
	120–350	17(24.64)	52 (75.36)			25(34.72)	47(65.28)			32(43.84)	41(56.16)		
	370–900	8(13.79)	50(86.21)			31(52.54)	28(47.46)			30(50.85)	29(49.15)		
	1000–3000	16(29.63)	38(70.37)			24(42.86)	32(57.14)			28(50.00)	28(50.00)		
Ownership of Car/Motor	No	28(14.21)	169(85.79)	7.09	0.008	52(25.74)	150(74.26)	11.15	0.001	56(27.45)	148(72.55)	22.15	0.000
	Yes	44(25.14)	131(74.86)			75(41.90)	104(58.10)			92(50.83)	89(49.17)		
History of pregnancy	No	26(22.61)	89(77.39)	1.13	0.288	42(35.00)	78(65.00)	0.22	0.640	47(38.84)	74(61.16)	0.01	0.913
	Yes	46(17.90)	211(82.10)			85(32.57)	176(67.43)			101(38.26)	163(61.74)		
Previous pregnancy complications	No	59(18.27)	264(81.73)	1.86	0.172	115(34.64)	217(65.36)	1.98	0.159	133(39.82)	201(60.18)	2.03	0.155
	Yes	13(26.53)	26(73.47)			12(24.49)	37(75.51)			15(29.41)	36(70.59)		
Current pregnancy complications	No	57(17.48)	269(82.52)	5.91	0.015	113(33.63)	223(66.37)	0.11	0.736	129(38.05)	210(61.95)	0.18	0.671
	Yes	15(32.61)	31(67.39)			14(31.11)	31(68.89)			19(41.30)	27(58.70)		
Parity	One Child	33(23.57)	107(76.43)	6.42	0.093	51(35.17)	94(64.83)	0.36	0.949	62(42.47)	84(57.53)	2.56	0.464
	Two children	17(18.28)	76(81.72)			30(32.26)	63(67.74)			33(35.11)	61(64.89)		
	Three Children	16(21.92)	57(78.08)			24(32.00)	51(68.00)			25(32.89)	51(67.11)		
	Four and more	6(9.09)	60(90.91)			22(32.35)	46(67.65)			28(40.58)	41(59.42)		
Health status during recent pregnancy	Excellent	7(23.33)	23(76.67)	2.86	0.413	11(36.67)	19(63.33)	2.41	0.491	12(40.00)	18(60.00)	3.22	0.358
	Very good	30(21.90)	107(78.10)			49(35.00)	91(65.00)			49(34.51)	93(65.49)		
	Good	26(15.57)	141(84.43)			51(29.65)	121(70.35)			67(38.73)	106(61.27)		
	Poor	9(23.68)	29(76.32			16(41.03)	23(58.97)			20(50.00)	20(50.00)		
Transport to facility	Walking	11(5.47)	190(94.53)	63.12	0.000	10(10.87)	82(89.13)	42.05	0.000	13(7.30)	165(92.70)	177.40	0.000
	Public transport	42(43.75)	54(56.25)			85(49.13)	88(50.87)			109(81.34)	25(18.66)		
	Personal means	19(25.33)	56(74.67)			32(27.59)	84(72.41)			26(35.62)	47(64.38		
Days after current birth	0-5days			-	-			-	-	15(21.43)	15(78.57)	12.65	0.005
	6-10days									74(39.15)	115(60.85)		
	11–39 days									31(49.21)	32(50.79)		
	40 and more days									28(44.44)	35(55.56)		
Mode of delivery	Vaginal			-	-	108(33.13)	218(66.87)	0.0425	0.837			-	-
	C-section					19(34.55)	36(65.45)						
Total		72(19.35)	300(80.65)			127(33.33)	254(66.67)			148(38.44)	237(61.56)		

Table 4 presents the results of the three logistic regression models that assessed the socio-economic and demographic characteristics associated with bypassing PHC facilities for ANC (model 1); delivery (model 2) and PNC (model 3). The three models are all statistically significant (Prob > chi2 = 0.000). As shown in Table 3, compared to those residing in the Jirapa Municipality, the Wa Municipal residents were about eight times more likely to bypass their PHC facilities for ANC (AOR = 8.51; *P* < 0.002; CI [2.19–33.03]), about 20 times more likely to bypass their PHC facilities for delivery (AOR = 20.20; *P* < 0.001; CI [6.93–58.89]and 17 times more likely to bypass their PHC facilities for PNC (AOR = 16.66; *P* < 0.001; CI [4.39–63.30]). Compared to the Walas, the Dagaabas were about twice more likely to bypass their PHC facilities for delivery (AOR = 2.09; *P* < 0.034; CI [1.06–4.12]) and about thrice more likely to bypass for PNC (AOR = 2.73; *P* < 0.016; CI [1.21–6.16]). Those from the other ethnicities were also about four times and seven times significantly more likely to bypass their PHC facilities for delivery (AOR = 3.78; *P* < 0.000; CI [1.79–7.97]) and PNC (7.39; *P* < 0.000; CI [2.60–21.05]) respectively compared to the Walas. In terms of education, only those who attained tertiary education were significantly more likely (AOR = 6.50; *P* < 0.017; CI [1.40–30.23]) to bypass their PHC facilities for ANC than those who have never had any formal education. Those who owned cars and/ motorbikes were about two times (AOR = 2.29; *P* < 0.035; CI [1.06–4.94]) and three times (AOR = 2.68; *P* < 0.016; CI [1.20–5.99]) more likely to bypass their PHC facilities for ANC and PNC, respectively. Those who suffered any pregnancy complications during their recent pregnancies were also about three times (AOR = 2.82; *P* < 0.028; CI [1.12–7.15]) more likely to bypass PHC facilities for ANC. Compared to those who walked to the health facilities, those who used public transport were statistically significantly more likely to bypass their PHC facilities for ANC (AOR = 10.41; *P* < 0.000; CI [4.39–24.65]) and PNC (AOR = 31.41; *P* < 0.000; CI [12.08–81.65]); and those who used their personal means of transport were also significantly more likely to bypass their PHC facilities for ANC (AOR = 2.34; *P* < 0.092; CI [0.87–6.30]) and PNC (OR = 5.15; *P* < 0.008; [CI of 1.52–17.44]). Those mothers who gave birth within 11–39 days prior to the data collection, were also about four times (AOR = 4.20; *P* < 0.027; CI [1.18–15.0]) more likely to bypass their PHC facilities for PNC compared to those who gave birth within the past five days.

**Table 4:** Socio-Economic and Demographic Factors Associated with Bypassing of PHC Facilities for Maternal Care.

Socio-economic predictor	Categories	Model 1 (ANC)			Model 2 (Delivery)			Model 3 (PNC)		
		AOR	95 % CI	P>|z|	AOR	95 % CI	P>|z|	AOR	95 % CI	P>|z|
Municipality of residence	Jirapa	*Reference*								
	Wa	8.51	2.19–33.03	0.002	20.20	6.93–58.89	0.000	16.66	4.39–63.30	0.000
Nature of residential location	Urban	*Reference*								
	Peri-urban	0.75	0.25–2.30	0.618	0.35	0.14–0.88	0.026	0.95	0.30–3.00	0.931
	Rural	1.17	0.46–2.99	0.741	1.54	0.76–3.12	0.225	1.36	0.56–3.32	0.499
Age	16–25	*Reference*								
	26–35	1.44	0.61–3.45	0.406	0.97	0.51–1.86	0.935	1.04	0.39–2.75	0.941
	> 35	0.16	0.04–0.54	0.004	2.00	0.81–4.91	0.130	1.59	0.50 - 5.03	0.429
Ethnicity	Waala	*Reference*								
	Dagaaba	-	-	-	2.09	1.06–4.12	0.034	2.73	1.21–6.16	0.016
	Others	-	-	-	3.78	1.79–7.97	0.000	7.39	2.60 -21.05	0.000
Education	No formal	*Reference*								
	Primary	0.64	0.15–2.68	0.544	0.86	0.33–2.32	0.792	0.68	0.18–2.43	0.540
	JHS	1.79	0.58–5.56	0.312	1.32	0.56–3.10	0.527	0.94	0.26–3.41	0.923
	SHS	1.83	0.63–5.33	0.268	1.56	0.68–3.62	0.292	1.27	0.43–3.71	0.664
	Tertiary	6.50	1.40–30.23	0.017	1.42	0.43–4.72	0.564	0.78	0.09–6.58	0.817
Occupation	Formal sector	Reference								
	Vocation	3.11	0.81–11.98	0.098	0.46	0.09–2.25	0.340	0.66	0.07–5.72	0.703
	Trade	1.62	0.36–7.20	0.526	0.52	0.10–2.61	0.426	0.33	0.03–3.14	0.335
	Farming	1.15	0.19–6.83	0.875	0.36	0.07–1.91	0.233	0.33	0.04–2.96	0.320
	Unemployed	2.94	0.69–12.47	0.143	0.22	0.04–1.30	0.095	0.66	0.07–6.03	0.710
Monthly Mother’s income (GH₵)	Zero	*Reference*								
	10–100	-	-	-	0.58	0.24–1.41	0.234	0.33	0.12–0.90	0.031
	120–350	-	-	-	0.38	0.12–1.19	0.097	0.64	0.22–1.89	0.422
	370–900	-	-	-	0.47	0.14–1.55	0.214	0.33	0.08 1.42	0.137
	1000–3000	-	-	-	0.20	0.03–1.10	0.064	0.35	0.04–3.04	0.344
Ownership of Car/Motor	No	*Reference*								
	Yes	2.29	1.06–4.94	0.035	1.78	0.98–3.22	0.056	2.68	1.20–5.99	0.016
Pregnancy complications	No	*Reference*								
	Yes	2.82	1.12–7.15	0.028	-	-	-	-	-	-
Transport to facility	Walking	*Reference*								
	Public	10.41	4.39–24.65	0.000	2.35	0.94–5.89	0.068	31.41	12.08–81.65	0.000
	Personal	2.34	0.87–6.30	0.092	2.60	0.88–7.67	0.084	5.15	1.52–17.44	0.008
Days after current birth	0-5days	*Reference*								
	6-10days	-	-	-	-	-	-	1.55	0.54–4.46	0.413
	11–39 days	-	-	-	-	-	-	4.20	1.18–15.01	0.027
	40 + days	-	-	-	-	-	-	2.99	0.80–11.19	0.103
Constant		0.0017	0.00–0 0.02	0.000	0.026	0.00–0.28	0.002	0.004	0.00–0.07	0.000
Observations		372			381			385		
Prob > chi2		0.000			0.000			0.000		
Pseudo R2		0.3711			0.2868			0.5473		
Log pseudolikelihood		-114.93819			-172.96563			-116.11477		

On the other hand, compared to those residing in urban areas, the peri-urbans residents were statistically significantly less likely (AOR = 0.35; 95 %CI [0.14–0.88]) to bypass their PHC facilities for delivery services. Also, those in their late childbearing age (35+) were statistically significantly less likely (AOR = 0.16; 95 %CI [0.04–0.54]) to bypass their PHC facilities for ANC. In terms of income, only those who earned below 100 Ghana Cedis per month were significantly less likely (AOR = 0.33; 95 % CI [0.12–0.90]) to bypass their PHC facilities for PNC compared to those who earn zero income per month.

### The cost of bypassing PHC facilities for maternal health care

Table 5 illustrates the results of the two-sample student’s t-test of differences in means of the cost of ANC, delivery, and PNC between those who bypassed their PHC facilities and those who did not bypass. The results show that for all the cost items, there was a positive mean difference in healthcare cost between those who bypassed and those who did not. This implies that on average, those who bypassed their PHC facilities paid an extra cost for bypassing. This extra cost (mean difference) was statistically significant (*p* < 0.05) for the total costs of the three maternal care services and for their specific cost items with the exception of the mean difference in medical cost for ANC (*p* = 0.1135) and that of the other expenses for delivery (*p* = 0.07) that were statistically insignificant.

**Table 5:** The Cost of Bypassing PHC facilities for Maternal Care.

Cost item	Full sample		By-passers		Non by-passers		Difference in Means Mean (bypass)-Mean (non-bypass)		T-test	
	Mean *GH*₵^1^	95 % CI *GH*₵	Mean *GH*₵	95 %CI *GH*₵	Mean *GH*₵	95 %CI *GH*₵	Mean *GH*₵	95 %CI *GH*₵	t	P r (T > t)
ANC										
Transportation	12.46	10.06–14.87	24.5	18.50–30.50	9.58	7.05–12.10	14.92	9.01–20.83	4.96	0.0000
Living expenses	7.13	5.42–8.83	11.30	7.37–15.24	6.12	4.23–8.014	5.18	0.88–9.48	2.37	0.0092
Medical expenses	63.39	53.73–73.05	75.51	53.07–97.95	60.48	49.75–71.22	15.030	-9.40–39.45	1.21	0.1135
Other expenses	10.95	8.55–13.35	17.57	10.32–24.81	9.37	6.96–11.77	8.20	2.17–14.23	2.68	0.0039
Total	93.94	80.73-107.15	128.89	95.96–161.81	85.55	71.26–99.83	43.34	10.15–76.53	2.57	0.0053
Observations	372		72		300					
Delivery										
Transportation	22.00	18.33–25.67	30.59	23.87–37.31	17.71	13.41- 22.00	12.88	5.19–20.57	3.29	0.0005
Living expenses	14.66	11.90-17.42	24.49	18.57–30.41	9.75	7.02–12.48	14.74	9.07–20.41	5.11	0.0000
Medical expenses	44.95	35.14–54.75	97.52	73.03–122.00	18.66	12.52–24.80	78.86	59.61–98.10	8.06	0.0000
Other expenses	13.96	10.44–17.47	17.70	11.09–24.31	12.09	7.97–16.21	5.61	-1.83–13.06	1.48	0.07
Total	95.57	81.87 -109.27	170.30	140.64- 199.96	58.20	46.25–70.16	112.09	85.30–138.90	8.22	0.0000
Observations	381		127		254					
PNC										
Transportation	6.41	5.07–7.74	14.83	11.89–17.77	1.15	0.69–1.60	13.68	11.30–16.06	11.30	0.0000
Living expenses	4.13	2.92–5.35	9.82	6.98–12.67	0.59	0.09–1.08	9.24	6.92–11.56	7.83	0.0000
Medical expenses	32.43	23.07–41.79	77.78	55.35 - 100.21	4.11	2.20–6.02	73.67	55.89–91.46	8.15	0.0000
Other expenses	1.64	0.93–2.34	3.49	1.73–5.26	0.48	0.23–0.73	3.02	1.59–4.44	4.17	0.0000
Total	44.61	33.34–55.89	105.93	79.52–132.35	6.32	4.07–8.57	44.61	33.33–55.90	9.35	0.0000
Observations	385		148		237					

^1^Exchange rate of approximately 1US$= GH₵5.68 as at January, 2020

On average, those mothers who bypassed their PHC facilities to deliver at other facilities incurred an extra cost of GH₵112.09 (US$19.73). There was also an average cost of GH₵44.61(US$7.85) and GH₵43.34 (US$7.63) for bypassing PHC facilities for PNC and ANC, respectively. In terms of the various cost items, the major source of the cost of bypassing came from medical expenses on consultation, treatment, and medicines for delivery (GH₵78.86 [US$13.88]) and PNC (GH₵73.67[US$12.97]). On average, those who bypassed their PHC facilities incurred an extra transportation cost of GH₵14.92 (US$2.63) for ANC, GH₵12.88 (US$2.27) for delivery and GH₵13.68 (US$2.41) for PNC.

## Discussion

The study assessed the prevalence of bypassing PHC facilities for maternal healthcare, and the socio-economic factors and financial costs associated with the phenomenon of bypassing PHC facilities for maternal healthcare in the northwestern part of Ghana. The results showed that the rate of bypassing PHC facilities ranges from 19.35 % for ANC, 33.33 % for delivery to 38.44 % for PNC. These findings confirm the existence of bypassing PHC facilities for maternal healthcare in the study area, which is in line with the findings of other studies in SSA [3, 5, 24, 26] and in Asia [4, 6, 10, 23]. Our rates of bypassing are, however, slightly lower than those (37.7- 70 %) reported by a majority of other studies in LMICs [3, 5, 24]. It is only our rate of bypassing for PNC (38.44 %) that fell within the existing prevalence range of bypassing facilities for maternal healthcare in LMICs.

The differences in the prevalence of bypassing PHC facilities between our study and that of others maybe because of computational variations. In our study, a mother was only considered to have bypassed if she consistently did not use her PHC facilities for all the times she sought maternal care and was not referred to those destination facilities. However, when we recalculated the bypassed rate in our study based on women not visiting their PHC facilities for at least once, as the case in other studies, our results, especially that for ANC (51.08 %), became comparable with that of the existing studies [4, 26]. Also, the differences in the rates may as well be attributed to contextual variations. Majority of the existing studies on bypassing for maternal care were from other parts of Africa (East Africa) [2, 3, 5] and Asia [4, 10, 23] but not in West Africa. The only previous study on bypassing in Ghana, although not focused on maternal care, reported a bypass rate of 33.9 % [32] which appears closer to some of the rates that were recorded in our study. It is therefore, reasonable to conclude that, the findings from our study are consistent with the bypass rates for health care services within the Ghanaian context. However, given that our study was the first to estimate the prevalence of bypassing PHC facilities for maternal care, there is the need for similar studies across other parts of Ghana to establish a comprehensive national perspective of the phenomenon of bypassing PHC facilities for maternal healthcare.

This evidence of bypassing is an indicator of poor acceptability of the maternal healthcare services delivered by the bypassed PHC facilities [38]. By implication, the findings of this study question the practical feasibility of implementing the Ghana healthcare gatekeeper and referral policy. Existing studies have revealed that a lot of PHC facilities in Northern Ghana[21] and elsewhere [19, 20] are less attractive to women seeking maternal care because of lack of midwives, maltreatment of clients, and poor experience of women with the existing healthcare referral system. It is against this background that some scholars have argued that bypassing PHC to higher level facilities may be important for women to obtain quality maternal healthcare[18]. To improve upon the relevance and practical feasibility of the current Ghana healthcare gatekeeper and referral system, deliberate policy reforms aimed at strengthening the service delivery capacities of PHC facilities are needed. With the high rates of bypassing PHC facilities in Ghana and elsewhere, there is also the need to assess the capacities of higher level healthcare facilities within LMICs to function effectively in the light of the overcrowding and increased workloads that are associated with bypassing PHC facilities [22]. The specific reasons why women bypass PHC facilities, particularly those relating to supply side challenges of health service delivery, also need to be explored in future research as that was beyond the scope of this current study. However, the demand side characteristics of the women that were significantly associated with the phenomenon of bypassing PHC facilities are discussed as follows:

Our study found that the municipality of which the mother resided was statistically significantly associated with bypassing PHC facilities for all maternal healthcare services. Women in the Wa Municipality (urban) were more likely to bypass their PHC facilities for all maternal health care services as compared to women in the Jirapa Municipality (rural). This may be as a result of the existence of a variety of health care facilities (higher facilities, mission/private) in the predominantly urban Wa Municipality as compared to the predominantly rural Jirapa Municipality where the residents had limited choice options in terms of variety of different types of healthcare facilities. This explanation is consistent with the extant literature as in Kenya and India, women living in urban catchment areas with different levels of healthcare facilities were also more likely to bypass their nearest PHC facilities than those living in the peripherals [5, 6].

Furthermore, our finding of ethnicity being a predictor of bypassing PHC facilities for delivery and PNC is in tandem with the Anderson [29] behavioral model that postulates that ethnicity as a socio-structural predisposing factor can influence people’s propensity to use health services. It is not very clear why the Dagaabas were more likely to bypass their PHC facilities. However, a plausible explanation could be that since they constituted most of the population in the study area, the Dagaabas may have certain advantages, including networks with higher level facilities, that enabled them to bypass their PHC facilities compared to those from the minority ethnic groups. This plausible interpretation is in line with that of a study in Nepal that found that women from an advantaged ethnic group were more likely to bypass for maternal health care than those from the disadvantaged ethnic group [4].

Higher educational attainment equips women with better knowledge and awareness of maternal health care issues [2, 5, 30]. Women who have had higher levels of education may demand higher level of pregnancy related services and hence, are more likely to bypass the lower level facilities where these services are not always available for ANC [6, 10, 23]. It was, therefore, not surprising that compared to those with lower levels of education, those women that attained tertiary education were more likely to bypass their PHC facilities for ANC. On the contrary, some studies found no relationships between educational levels and bypassing [3, 4].

Ownership of means of transport is an enabling factor in the use of health care services [29]. Due to the longer distance that is usually associated with bypassing facilities [2], bypassing is most likely to be facilitated by the use of a car/motorbike and other public means of transport either than walking to the facility [10]. This explains our findings that mothers who owned and/ used their personal cars/motorbikes or public means of transport were significantly more likely to bypass their PHC facilities for ANC and PNC compared to those who did not own such means of transport and hence had to walk to the healthcare facilities to seek care.

According to Rosenstock [39], the severity of a health condition compels people to take drastic action to reduce the impact of the condition. As would, therefore, be expected, mothers who had complications during their current pregnancy were three times significantly more likely to bypass their PHC facilities to higher level facilities for ANC. This finding conforms with that of other researches in SSA [25] and beyond [4, 6, 10, 23]. Besides, those mothers who delivered between 11 and 39 days and were receiving PNC were also significantly more likely to bypass their nearest PHC facilities for the service. This may be explained by the fact that none of the required PNC visits is scheduled within the 11th -39th day after birth. Hence, there is the likelihood that mothers who sought for PNC within this period were defaulters who were visiting either late or earlier than their schedules and as a result could not go to their closest (scheduled) PHC facilities because of fear of been questioned by the providers hence bypassing their PHC facilities.

The seemingly inverse associations of income and age with bypassing are the most difficult to explain. The finding that compared to those in the no income group, those in the least income group (those who earned a maximum of GH₵ 100 [US$17.60]) were significantly less likely to bypass their PHC facilities for PNC seems counter-intuitive. This is because the theoretical expectation would be that the little earnings would have served as an enabling factor for bypassing [29]. However, given that the associations between bypassing and the more higher income groups were all statistically insignificant, we do not think that income indeed can be an adequate predictor of bypassing in this study. We suspect that this least income category of women were petty traders and farmers whose livelihood activities were around their places of residence and who would not be willing to travel to distant locations for PNC unlike the no income group who were likely to be unemployed and could have time to travel to distant facilities for PNC. Also, unlike in other studies where older women were more likely to bypass their PHC facilities [3, 4, 26] or no statistically significant associations were reported between age and bypassing PHC facilities [2, 23], our study revealed that those in their late child bearing age were rather less likely to bypass their PHC facilities for ANC than those in their early child bearing age. We argue that this is so because older women might have had previous experiences of childbirth and had already received a lot of information about pregnancy issues from their previous ANC visits and hence would not require/demand a lot of such information that would necessitate bypassing to higher level facilities.

Our study also established that bypassing PHC facilities for maternal healthcare was associated with a significantly higher cost. This finding is in line with that of others across the globe which also reported an additional cost of bypassing for maternal health care [2, 3, 24]. Meeting this extra cost could be very catastrophic as bypassers are sometimes compelled to sell off household assets or borrow to pay for such cost [24, 31]. In line with the findings of Kruk et al. [3], the mothers who bypassed for care paid considerably higher cost for transportation compared to their counterparts who did not bypass. This additional cost comes as a results of the fact that, most of the bypassed facilities are located farer from the mother’s home of residence hence the mothers are required to pay higher transport fares to reach such facilities [25]. Mothers often use private cars or hired taxis at high cost to the bypassed facilities to ensure the safety of both the mother and child [10, 23, 24]. Our findings are also in line with existing literature that, both the mother and their informal caregivers incurred higher living expenses on feeding and accommodation at the bypassed facilities than at the PHC facilities [23]. Interestingly, the mothers who bypassed their PHC facilities paid significantly more for the cost of medical services (consultation, treatment, and medicines) for delivery and PNC but not for ANC. Due to the free maternal healthcare policy [11], women who bypassed for ANC, provided they went to facilities with NHIS accreditation would not be expected to pay extra for medicine. However, the extra medical cost incurred by bypassers for delivery and PNC could be explained in line with what Kruk et al. [3] asserted that, for those services, bypassers often visit mission or private facilities where cost of medical services is considerably higher due to out-of-pocket payments at such facilities. Despite maternal healthcare being officially free of charge in Ghana, our study and that of Dalinjong et al. [13] reported the existence of informal and other non-receipted expenses, which in our study, was disproportionally incurred by the bypassers than those who did not bypass their PHC facilities. This highlights the need for the Ghana NHIS to adopt an innovative mechanism of paying for some of the non-medical costs, including transportation cost, of seeking maternal healthcare.

## Limitations of the study

First, the study was biased towards mothers who did attend PNC after delivery. This is because the study was a facility-based survey although a household survey would have given every woman who was within the target population the opportunity to be part. Also, the study did not do a geographic information system analysis to establish if the women’s reported PHC facilities were indeed the nearest PHC facilities to them. Our verification of the veracity of the self-reported PHC facilities was only based on comparing the woman’s location vis-à-vis her reported PHC facility with a list of health facilities and their catchment areas obtained from the Regional Health Directorate. Our study did not also cover the opportunity cost of bypassing which can be very substantial within LMICs. Future research should therefore undertake a household survey of a representative sample and avoid the limitations inherent in our study. Despite these limitations, this study remains the first within the Ghanaian context to examine the phenomenon of bypassing PHC facilities for maternal healthcare and hence provides an essential reference for further studies on this subject matter.

## Conclusions

 The study revealed evidence of bypassing PHC facilities for maternal health care services in the study area. This evidence of bypassing is an indication of poor acceptability of the maternal care services delivered by the bypassed PHC facilities and practical challenges of enforcing the existing GHS recommended gatekeeper system. The municipality of residence, ethnicity, tertiary education, ownership of cars and/motorbikes, pregnancy complications, means of transport used to the health facility, days after the current birth, nature of residential location, age and income, were significantly associated with bypassing PHC facilities. There is an extra cost in financial terms for bypassing which comes because of transportation, feeding and accommodation, medical costs and other non-receipted costs. Addressing the phenomenon of bypassing and its associated cost, will require effective policy reforms aimed at strengthening the service delivery capacities of PHC facilities. We recommend that the Ministry of Health and Ghana Health Service should organize stakeholder engagement and sensitizations campaigns for women and the communities at large on the consequences of bypassing PHC facilities for maternal health care. Future research, outside healthcare facility settings, is also required to understand the specific supply-side factors influencing bypassing of PHC facilities for maternal healthcare within the study area.

## Data Availability

The datasets generated and/or analysed during the current study are not publicly available because the data was purposively collected for the MPhil thesis of the first author but are available from the corresponding author on reasonable request.

## Abbreviations

ANC: Antenatal care
CHPS: Community-based Health Planning and Services
GH₵: Ghana Cedis
GHS: Ghana Health Service
LMICs: Low- and middle-income countries
NHIS: National Health Insurance Scheme
PHC: Primary health care
PNC: Postnatal care
SSA: Sub-Saharan Africa
UWRHD-GHS: Upper West Regional Health Directorate, Ghana Health Service
